# p53 protein regulates the effects of amifostine on apoptosis, cell cycle progression, and cytoprotection

**DOI:** 10.1038/sj.bjc.6600779

**Published:** 2003-03-04

**Authors:** E J Lee, M Gerhold, M W Palmer, R D Christen

**Affiliations:** 1 Department of Medicine and Cancer Center, 0058, University of California San Diego, 9500 Gilman Drive, La Jolla, CA 92093-0058, USA; 2 Department of Oral Pathology, Oral Cancer Research Institute, Yonsei University College of Dentistry, 134 Shinchon-Dong, Seaodaemun-ku, Seoul, 120-752, Korea

**Keywords:** amifostine, p53 protein, p21 protein, cell cycle arrest, apoptosis, cytoprotection

## Abstract

To determine the role of p53 protein on the cellular effects of amifostine, we used molecularly engineered HCT116 colon cancer cells in which the *p53* gene was inactivated by targeted homologous recombination or p53 protein was degraded by high-level expression of papillomavirus E6 protein. Amifostine induced a G1 arrest and protected against paclitaxel toxicity in p53-proficient but not in p53-deficient cells. In the absence of p53 protein, amifostine enhanced the cytotoxicity of paclitaxel. In addition, treatment of HCT116 cells with amifostine alone resulted in apoptotic cell death. Compared with p53-deficient cells, p53-proficient cells exhibited low-level resistance to amifostine-induced apoptosis. Amifostine induced the expression of p53 protein in p53-proficient cells and the expression of p21 protein in both p53-proficient and -deficient cells. These findings indicate that amifostine-induced G1 arrest and cytoprotection are mediated via a pathway that is dependent on p53 protein and that amifostine-induced expression of p21 protein is not sufficient to sustain a G1 arrest or to mediate cytoprotection. In addition, these findings identify p53 protein as a mechanism of resistance to amifostine-induced apoptosis.

Amifostine, designated WR-2721, was originally synthesised at the Walter Reed Army Institute of Research in the 1950s to protect military personnel from nuclear radiation. Amifostine protects cells from cytotoxic damage by scavenging oxygen-free radicals caused by radiation and radiomimetic drugs and by binding to reactive nucleophiles, which have the potential to react with DNA (van der Vijgh and Peters, 1994).

Amifostine has demonstrated broad-spectrum cytoprotection against myelotoxicity, nephrotoxicity, xerostomia, and mucositis associated with various chemotherapy and radiation regimens (Capizzi and Oster, 2000). Large comparative clinical trials of amifostine in patients with advanced ovarian cancer (Kemp *et al*, 1996), rectal cancer (Liu *et al*, 1992), and head and neck cancer (Brizel *et al*, 2000) have been completed. These trials have shown that amifostine protects against the cytotoxic effects of cisplatin, cyclophosphamide, and radiation therapy. In addition, amifostine protects against xerostomia in patients with head and neck cancer treated with radiation therapy and in patients with thyroid cancer treated with high-dose radio-iodine (Bohuslavizki *et al*, 1998; Buntzel *et al*, 1998). Recent observations also indicate that amifostine improves cytopenia in patients with myelodysplastic syndromes (List *et al*, 1997,^1998^).

WR-1065, the active metabolite of amifostine, protects cells from cytotoxic damage by scavenging oxygen-free radicals generated by radiation and anthracyclines and by binding to highly reactive nucleophiles and thus preventing nucleophiles from reacting with DNA (Treskes and van der Vijgh, 1993). The sulphydryl group of WR-1065 inactivates carbonium ions generated by alkylating agents and thereby prevents the formation of DNA crosslinks (DeNeve *et al*, 1988). Amifostine also protects against the cytotoxicity of paclitaxel, a cytoprotective effect that is not readily explained (Taylor *et al*, 1997).

In addition to its cytoprotective properties, amifostine has other interesting biologic effects. For instance, WR-1065 reduces the level of phosphorylation and inhibits the enzymatic activity of topoisomerase II (Grdina *et al*, 1994; Murley *et al*, 1997; Snyder and Grdina, 2000). Amifostine and its metabolite upregulate genes involved in cell proliferation, including c-*myc* and thymidine kinase (Woloschak *et al*, 1995; Liu *et al*, 1997). In mouse fibroblasts and breast cancer cells, amifostine and its metabolite WR-1065 have been shown to activate p53 protein, to induce the expression of the cyclin-dependent kinase inhibitor p21, and to arrest cells at the G1/S transition via a p53-dependent pathway (North *et al*, 2000). In addition, amifostine binds to the transcription factors nuclear factor-*κ*B, activator protein-1, and p53 protein, resulting in enhanced binding of these proteins to target regulatory DNA sequences and subsequent transactivation of downstream genes (Shen *et al*, 2001). Similarly, in a yeast functional assay, amifostine restored the transcriptional activity of p53 mutants (Maurici *et al*, 2001). In nontransformed cells, WR-1065 protects cells from the cytotoxic effects of paclitaxel in a p53-dependent manner (Shen *et al*, 2001). However, amifostine had no cytoprotective effect in transformed human tumour cells, suggesting that p53-dependent growth arrest is the basis for the protective effect of amifostine and that this pathway is abrogated in human tumours (Shen *et al*, 2001).

In the current study, we used two sets of p53-proficient and -deficient cell lines to determine the effect of p53 protein on amifostine-induced apoptosis, cell cycle arrest, and cytoprotection.

## MATERIALS AND METHODS

### Cell lines

Parental human colon carcinoma HCT116 cells were obtained from the American Type Culture Collection (ATCC, CCL 247). HCT116 cells contain a hemizygous mutation in hMLH1, resulting in a truncated, nonfunctional protein (Boyer *et al*, 1995). A subline complemented with chromosome 3, designated HCT116+ch3, was obtained from Drs CR Boland and M Koi (Koi *et al*, 1994). The chromosome 3-complemented cells were competent in DNA mismatch repair (Koi *et al*, 1994). HCT116 cells in which both *p53* alleles were deleted by targeted homologous recombination, designated HCT116/p53^−/−^, were obtained from Dr Bert Vogelstein (Bunz *et al*, 1999). HCT116 cells and HCT116+ch3 expressing papillomavirus E6, designated HCT116/E6 and HCT116+ch3/E6, were obtained from Drs A Boothman and M Meyers. These cell lines constitutively express high levels of human papillomavirus type-16 E6 protein, which stimulates the degradation of p53 protein via an ubiquitin-dependent pathway (Davis *et al*, 1998). All cell lines were maintained in Iscove's modified Dulbecco's medium (Irvine Scientific, Irvine, CA, USA) supplemented with 100 mM L-glutamine and 10% heat-inactivated foetal bovine serum. The chromosome-complemented lines were grown in medium supplemented with 400 *μ*g ml^−1^ geneticine (GIBCO BRL, Gaithersburg, MD, USA). HCT116/p53^+/+^ and HCT116/p53^−/−^ cells were maintained in McCoy's medium (Irvine Scientific, Irvine, CA, USA) supplemented with 100 mM L-glutamine and 10% heat-inactivated foetal bovine serum.

### Reagents

Amifostine was obtained from US Bioscience Corporation; a stock solution of 50 mg ml^−1^ was prepared in 0.9% NaCl and stored at 4°C. Paclitaxel was obtained from Sigma Chemical Co. (St Louis, MO, USA) and was dissolved in DMSO.

### Clonogenic assay

For clonogenic assays, 500 cells were seeded in plastic dishes containing complete media. The cells were allowed to attach overnight, and on the next day, cells were exposed to amifostine at increasing concentrations for 24 h. Thereafter, the cells were washed with PBS and fresh, drug-free medium was added. After 10–14 days of incubation in humidified 5% CO_2_ at 37°C, cells were washed, fixed with methanol, and stained with 0.1% crystal violet. Cell clusters containing more than 50 cells were scored as a colony. Each experiment was performed a minimum of three times using triplicate cultures for each drug concentration. IC_50_ (concentration causing 50% inhibition of cell growth) values were estimated using log-linear interpolation.

### Cell cycle phase distribution

Approximately 1 × 10^6^–2 × 10^6^ cells were exposed to amifostine for 24 h. At various points in time after starting amifostine treatment, cells were harvested, washed twice with ice-cold PBS, and fixed in ice-cold 100% ethanol. Cells were counted and 10^6^ cells per sample were centrifuged, resuspended in 500 *μ*l of ice-cold PBS, and treated with 0.1 mg ml^−1^ RNAse A (Sigma Chemical Co.) at 37°C for 30 min. Propidium iodide (Molecular Probes, Eugene, OR, USA) at a final concentration of 50 *μ*g ml^−1^ was then added to the cell suspensions. After incubation on ice for 30 min, cells were analysed on a FACScan flowcytometer (Becton-Dickinson, San Jose, CA, USA). Multicycle AV Cell Cycle software (Phoenix Flow Systems, San Diego, CA, USA) was used to calculate the fraction of cells in each phase of the cell cycle as previously described (Dean and Jett, 1974).

### Quantitation of apoptotic cells

Cells were treated with amifostine for 24 h. At various points in time, floating and attached cells were harvested, centrifuged, resuspended in 100 *μ*l of PBS, and stained with acridine orange and ethidium bromide. Subsequently, cells were assessed for apoptotic morphology by supravital fluorescence microscopy. Cells were scored as apoptotic according to established morphologic criteria (McGahon *et al*, 1995).

### Western blot analysis

Logarithmically growing cells were treated with amifostine for 24 h. At various points in time after the beginning of exposure to amifostine, cells were lysed and proteins extracted in a buffer containing 0.15 M NaCl, 5 mM EDTA. 1% Triton X-100, 10 mM TRIS (pH 7.4), 5 mM DTT, 0.1 mM phenylmethylsulphonyl fluoride, and 5 mM epsilon-aminocaproic acid. Proteins were quantitated and fractionated by electrophoresis using 7.5 and 15% polyacrylamide gels (Precast Acrylamide Gels, Bio-Rad, Hercules, CA, USA). Proteins were transferred to polyvinylidene difluoride membranes (Immobilon, Millipore, Bedford, MA, USA). The membranes were blocked with 5% nonfat milk, 0.05% Tween for 1 h, washed with 0.05% Tween and then exposed to primary antibody overnight at 4°C. Mouse monoclonal anti-p21 (sc-817) and anti-p53 (sc-126) antibodies were obtained from Santa Cruz Biotechnology, Santa Cruz, CA, USA. Primary antibodies were diluted 1 : 400 in 5–10% nonfat milk. After washing, blots were exposed to horseradish peroxidase-conjugated anti-mouse antibodies (Amersham Life Science, Inc., Arlington Heights, IL, USA) at a dilution of 1 : 3000, and complexes were visualised by enhanced chemiluminescence.

## RESULTS

To investigate how p53 protein regulates the cellular response to amifostine, we used p53-proficient parental HCT116/p53^+/+^ and p53-deficient HCT116/p53^−/−^cells, in which both *p53* alleles were deleted by homologous recombination (Davis *et al*, 1998). HCT116 cells are genomically instable because of a homozygous mutation at the hMLH1 locus (Koi *et al*, 1994; Papadopoulos *et al*, 1994). As a result, HCT116 cells have accumulated multiple mutations, including mutations of the genes encoding the type II receptor for transforming growth factor *β* 1, the insulin-like growth factor II receptor, and the proapoptotic gene BAX (Carethers and Pham, 2000). In addition, defective expression of hMLH1 in HCT116 cells results in alterations in the G2/M cell cycle checkpoint following radiation (Hawn *et al*, 1995). In order to control for spurious effects that are a result of genomic instability and alterations in cell cycle progression, we also used a mismatch repair-proficient HCT116 subline complemented with chromosome 3. HCT116+ch3 cells are genomically stable and have an intact G2/M checkpoint following radiation (Hawn *et al*, 1995; Carethers and Pham, 2000). In HCT116+ch3 cells, p53 was inactivated by constitutive high-level expression of the human papillomavirus type 16 E6 gene, which stimulates the degradation of p53 protein (Davis *et al*, 1998).

### Effect of p53 protein on amifostine toxicity

**Figure 1:**
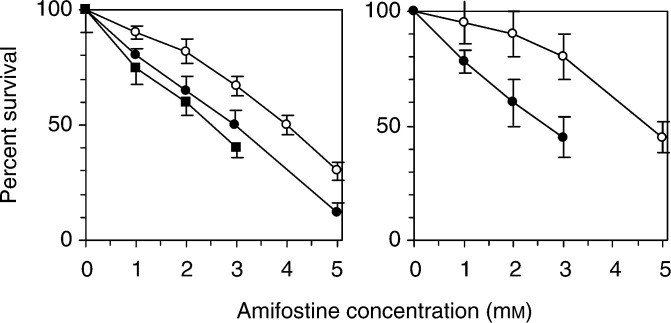
Effect of p53 protein on amifostine toxicity. HCT116 cells were exposed to amifostine for 24 h, and dose–response curves were generated by clonogenic assay. Left panel: (○), HCT116/p53^+/+^ cells; (•), HCT116/p53^−/−^ cells; (▪), HCT116/E6 cells. Right panel: (○), HCT116+ch3 cells; (•), HCT116+ch3/E6 cells. Data points represent mean±s.d. of at least three independent experiments performed with triplicate cultures.

Clonogenic assays were used to determine the effect of p53 protein on the sensitivity of HCT116 colon cancer cells to amifostine (Figure 1

). After allowing cells to attach overnight, p53-proficient and -deficient HCT116 cells were exposed to increasing concentrations of amifostine for 24 h. The number of colonies was assessed after 10–14 days. In HCT116 cells, deletion of the *p53* gene by targeted homologous deletion resulted in a 1.4±0.2-fold increase in amifostine sensitivity, as quantitated by the ratio of the IC_50_ values (mean±s.d., *n*=3, *P*<0.05 by a two-sided *t*-test). Similarly, degradation of p53 protein by E6 resulted in a 1.6±0.2-fold increase in amifostine sensitivity (mean±s.d., *n*=3, *P*<0.05 by a two-sided *t*-test). In mismatch repair-proficient HCT116+ch3 cells, degradation of p53 protein by E6 resulted in a 1.9±0.2-fold increase in sensitivity to amifostine (mean±s.d., *n*=3, *P*<0.05 by a two-sided *t*-test).

### Effect of p53 protein on amifostine-induced apoptosis

**Figure 2:**
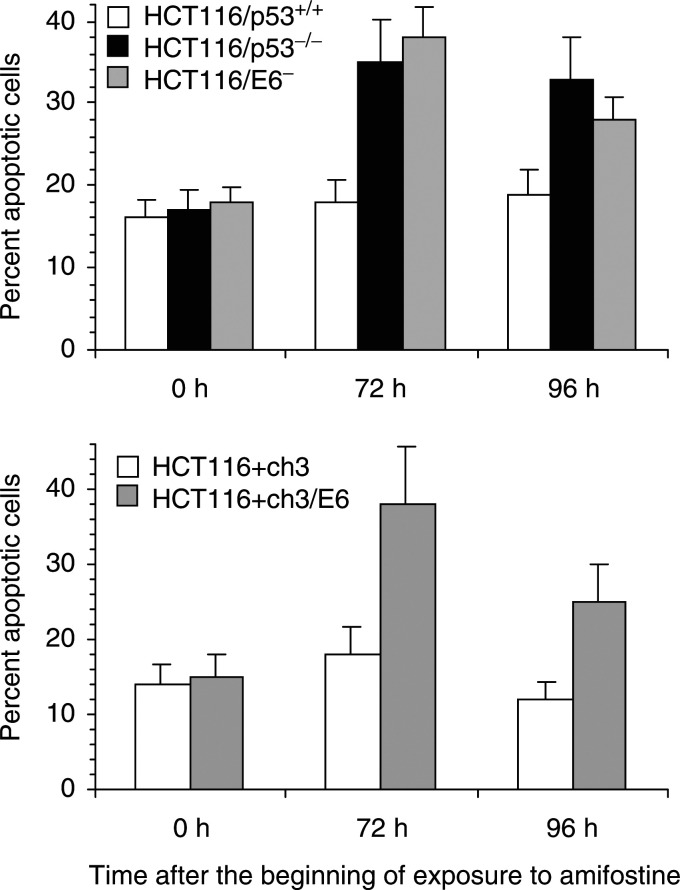
Effect of p53 protein on amifostine-induced apoptosis. HCT116 cells were exposed to amifostine for 24 h, and the fraction of apoptotic cells was determined by supravital fluorescence microscopy at 72 and 96 h after the beginning of exposure to amifostine. Upper panel: white columns, HCT116/p53^+/+^ cells; black columns, HCT116/p53^−/−^ cells; shaded columns, HCT116/E6 cells. Lower panel: white columns, HCT116+ch3 cells; shaded columns, HCT116+ch3/E6 cells. Columns and bars represent mean±s.d. of three independent experiments each performed with triplicate cultures.

Supravital fluorescence microscopy was used to quantitate apoptotic cells (Figure 2

). HCT116/p53^+/+^, HCT116/p53^−/−^, and HCT116/E6 cells were exposed to 3.8 mM amifostine for 24 h, a schedule that corresponded to an IC_50_ in HCT116/p53^+/+^ cells. HCT116+ch3 and HCT116+ch3/E6 cells were exposed to 4.9 mM amifostine for 24 h, which corresponded to an IC_50_ in HCT116+ch3 cells. The fraction of apoptotic cells was determined at 72 and 96 h after the beginning of exposure to amifostine. Equimolar concentrations of amifostine are expected to result in the same extent of cellular injury in cells that differ only with respect to p53 status. Therefore, any differential effect in amifostine-induced apoptosis is conjectured to be the result of p53-mediated, differential processing of cellular damage. In HCT116 cells, deletion of the *p53* gene by targeted recombination resulted in an increase in amifostine-induced apoptosis by 1.9±0.2- and 1.8±0.2-fold at 72 and 96 h after the beginning of exposure to amifostine, respectively (mean±s.d., *n*=3, *P*<0.05 by a two-sided *t*-test for the comparison of p53-proficient *vs* -deficient cells). Similarly, degradation of p53 protein by E6 resulted in an increase in amifostine-induced apoptosis by 2.1±0.2-fold and 1.5±0.2-fold at 72 and 96 h after the beginning of exposure to amifostine, respectively (mean±s.d., *n*=3, *P*<0.05 by a two-sided *t*-test for the comparison of p53-proficient *vs* -deficient cells). In mismatch repair-proficient HCT116+ch3 cells, E6-mediated degradation of p53 protein resulted in an increase in amifostine-induced apoptosis by 1.8±0.2- and 2.0±0.2- at 72 and 96 h, respectively (mean±s.d., *n*=3, *P*<0.05 by a two-sided *t*-test for the comparison of p53-proficient *vs* -deficient cells).

### Effect of p53 protein on cell cycle arrest induced by amifostine

**Figure 3:**
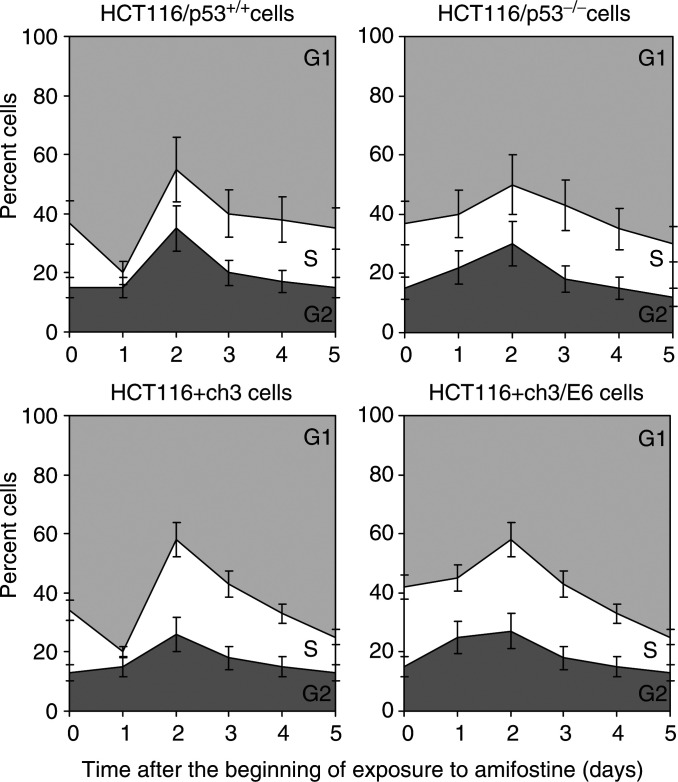
Effect of p53 protein on cell cycle arrest induced by amifostine. HCT116 cells were exposed to amifostine for 24 h and cell cycle phase distribution was determined by flowcytometry. Data points represent mean±s.d. of three independent experiments.

HCT116/p53^+/+^ and HCT116/p53^−/−^ cells were exposed to 3.8 mM amifostine for 24 h, which corresponded to an IC_50_ in HCT116/p53^+/+^ cells. HCT116+ch3 and HCT116+ch3/E6 cells were exposed to 4.9 mM amifostine for 24 h, which corresponded to an IC_50_ in HCT116+ch3 cells. In p53-proficient HCT116/p53^+/+^ and HCT116+ch3 cells, treatment with amifostine caused a G1 arrest that peaked at 24 h (Figure 3

). However, in p53-deficient HCT116/p53^−/−^ and HCT116+ch3/E6 cells, amifostine failed to cause a G1 arrest, indicating that the G1 arrest induced by amifostine is dependent on p53 protein. Amifostine also caused a G2/M arrest, which occurred in both p53-proficient and -deficient cells, indicating that the G2/M arrest is mediated via a pathway that is independent of p53 protein.

### Effect of p53 protein on amifostine-induced cytoprotection

**Figure 4:**
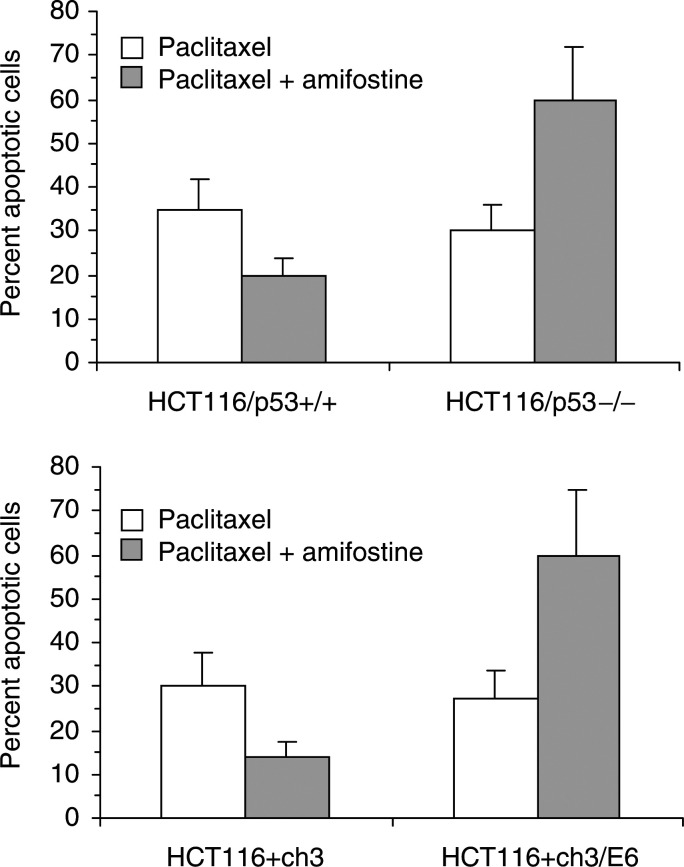
Effect of p53 protein on amifostine-induced cytoprotection. HCT116 cells were exposed to paclitaxel for 24 h in the absence or presence of amifostine. The fraction of apoptotic cells was determined by supravital fluorescence microscopy at 48 h after the beginning of drug exposure. Open columns, cells treated with paclitaxel alone; shaded columns, cells treated with paclitaxel and amifostine. Columns and bars represent mean±s.d. of three independent experiments.

To determine whether p53 protein regulates the cytoprotective properties of amifostine, we investigated the effect of amifostine on paclitaxel toxicity in p53-proficient and -deficient cells (Figure 4

). HCT116 cells were exposed to 20 nM paclitaxel for 24 h in the absence or presence of amifostine. Amifostine-treated cells were pretreated with 100 *μ*M amifostine for 30 min and subsequently exposed to amifostine and paclitaxel for 24 h. At this concentration, amifostine is nontoxic; however, even at low concentrations, amifostine has important biologic effects, such as induction of p53 protein, a finding that occurs at concentrations as low as 50 *μ*M (North *et al*, 2000). At 48 h after the beginning of drug exposure, cells were stained with acridine orange and ethidium bromide, and the fraction of apoptotic cells was determined by supravital fluorescence microscopy. Amifostine protected p53-proficient HCT116/p53^+/+^ and HCT116+ch3 cells from the cytotoxicity of paclitaxel and reduced paclitaxel-induced apoptosis by 1.75±0.2-fold (mean±s.d., *n*=3, *P*<0.05 by a two-sided *t*-test) and 2.0±0.2-fold (*P*<0.05), respectively. However, amifostine failed to protect p53-deficient cells from the cytotoxicity of paclitaxel, indicating that the cytoprotective effect of amifostine is p53-dependent. Interestingly, in p53-deficient HCT116/p53^−/−^ and HCT116+ch3/E6 cells, amifostine enhanced paclitaxel-induced apoptosis by 2.1±0.2-fold (mean±s.d., *n*=3, *P*<0.05 by a two-sided *t*-test) and 2.2±0.2-fold (*P*<0.05), respectively.

### Effect of amifostine on the expression of p53 protein and p21 protein

**Figure 5:**
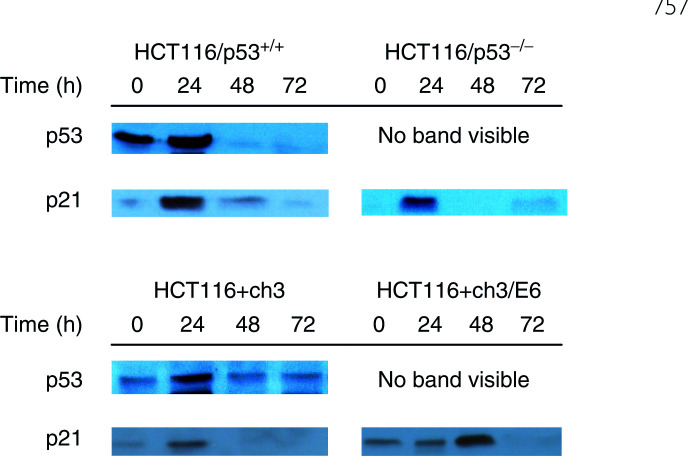
Effect of amifostine on the expression of p53 protein and p21 protein. HCT116 cells were exposed to amifostine for 24 h and cellular proteins were analysed by Western blotting at various points in time after the beginning of exposure to amifostine. Amifostine induced the expression of p53 protein in p53-proficient cells and the expression of p21 protein in p53-proficient and -deficient cells.

To further investigate the role of p53 protein and p21 protein on amifostine-induced apoptosis and cell cycle progression, we determined the effect of amifostine on the expression of p53 protein and p21 protein in p53-proficient and -deficient cells (Figure 5

). HCT116/p53^+/+^ and HCT116/p53^−/−^cells were exposed to 3.8 mM amifostine for 24 h, which corresponded to an IC_50_ in HCT116/p53^+/+^ cells. HCT116+ch3 and HCT116+ch3/E6 cells were exposed to 4.9 mM amifostine for 24 h, corresponding to an IC_50_ in HCT116+ch3 cells. In p53-proficient cells, amifostine caused a rapid induction of p53 protein, which, like the amifostine-induced G1 arrest, peaked at 24 h.

In addition, amifostine caused a rapid induction of p21 protein. Induction of p21 protein occurred in both p53-proficient and -deficient cells, indicating that the induction of p21 protein in response to amifostine is independent of p53 protein. In p53-deficient cells, amifostine induced the expression of p21 protein without causing a G1 arrest, indicating that overexpression of p21 protein in response to amifostine was not sufficient to arrest cells in the G1 phase of the cell cycle.

## DISCUSSION

The prodrug amifostine and its active metabolite WR-1065 have been shown to have differential effects on malignant *vs* nonmalignant tissue. For instance, amifostine protects normal tissues from the toxic effects of ionising radiation and chemotherapeutic agents, but has either no effect or enhances the antitumour effects of these agents (van der Vijgh and Peters, 1994; Taylor *et al*, 1997; Capizzi, 1999a; Shen *et al*, 2001). The mechanisms by which amifostine preferentially protects normal cells are poorly understood. Selective protection of normal tissue by amifostine has been associated with activation of a p53-dependent pathway, and amifostine has been shown to bind to p53 protein and to enhance transactivation of downstream genes (Shen *et al*, 2001).

We now report on the effects of p53 protein on the cellular pharmacology of amifostine in two sets of matched HCT116 cell lines that differ in p53 status. In the first set, the *p53* gene was deleted by homologous recombinant deletion; in the second set, the p53 protein was degraded by high-level expression of human papillomavirus E6 protein. In both sets of HCT116 cell lines, amifostine selectively protected p53-proficient cells from the cytotoxicity of paclitaxel. Since normal tissue is p53-proficient, this finding could, at least in part, explain the observation that amifostine protects normal tissues from the toxic effects of ionising radiation and chemotherapeutic agents but fails to protect malignant tissue, which is often p53-deficient. Many solid tumours differ from nonmalignant tissue in that they express defective forms of p53 protein itself or of the pathways downstream of p53 protein (Sherr and Weber, 2000).

Amifostine is a phosphorylated aminothiol prodrug that is dephosphorylated by membrane-bound alkaline phosphatase to form the active metabolite WR-1065, a free thiol that exhibits cytoprotective activity after being transported into the cell (van der Vijgh and Peters, 1994; Capizzi, 1999b). Preferential protection of normal cells has been associated with higher activity of membrane-bound alkaline phosphatase in normal endothelium and normal tissue, compared to neovascular endothelium and tumour tissue (Manheimer and Seligman, 1948; Romanul and Bannister, 1962). However, recent observations indicate that differences in the expression of alkaline phosphatase and cellular transport between normal and tumour tissue cannot entirely account for the selectivity with which amifostine protects normal tissue. For instance, the dephosphorylated metabolite of amifostine, WR-1065, which penetrates equally well into cultured cancer and noncancer cells, has also been found to have a preferential radioprotective effect on human diploid fibroblasts, as compared to HT-1080 fibrosarcoma cells (Zhang *et al*, 1992).

In spite of protecting p53-proficient cells from the cytotoxicity of paclitaxel, amifostine sensitised p53-deficient HCT116 cells to paclitaxel. Similarly, amifostine has been shown to sensitise CaCl melanoma cells to paclitaxel in a p53-dependent manner (Shen *et al*, 2001). In addition, amifostine has been shown to protect MRC-5 human lung fibroblasts from paclitaxel-induced cytotoxicity and to enhance paclitaxel cytotoxicity in A427 lung cancer cells (Taylor *et al*, 1997). Although A427 cells express p53 protein (Soini *et al*, 2001), these cells fail to arrest in response to DNA damage (Strobeck *et al*, 2001), indicating that the p53 pathway is inactive as a result of a defect in p53 protein itself or in a pathway downstream of p53 protein. The mechanisms by which amifostine enhances drug sensitivity in cells with a defective p53 pathway are not well understood. Interestingly, in amifostine-treated A427 cells, enhanced sensitivity to paclitaxel has been associated with increased formation of DNA single-strand breaks (Taylor *et al*, 1997).

In amifostine-treated cells, p53 protein also plays an important role in regulating progression through the cell cycle. Amifostine caused a G2 arrest in both p53-proficient and -deficient cells, indicating that the amifostine-induced G2 arrest is independent of p53 protein. In addition, amifostine induced the expression of p21 protein via a p53-independent pathway, a finding that is compatible with a role of p21 protein in amifostine-induced G2 arrest. Indeed, p21 protein has been shown to inhibit phosphorylation of Cdc2 and to enforce G2 arrest (Smits *et al*, 2000).

Amifostine also caused a G1 arrest, which, in contrast to the G2 arrest, occurred only in p53-proficient cells, indicating that the G1 arrest is dependent on p53 protein. In p53-proficient cells, both the G1 arrest and the induction of p53 protein peaked at 24 h after the beginning of exposure to amifostine, a finding consistent with the notion that the G1 arrest induced by amifostine is mediated via a p53-dependent pathway. Since amifostine induced the expression of p21 protein in both p53-proficient and -deficient cells but caused a G1 arrest only in p53-proficient cells, induction of p21 protein alone was not sufficient to sustain a G1 arrest after treatment with amifostine. In addition to regulating the transcription of the *p21* gene, p53 protein has been shown to regulate the transcription of genes encoding other downstream proteins, including other members of the Cip/Kip family, such as p27^Kip1^ and p57^Kip2^ (Shapiro and Harper, 1999). Like p21 protein, p27^Kip1^ and p57^Kip2^ proteins have also been shown to form complexes with cyclin E-CDK2 and to promote CDK2 inhibition and G1 arrest (Shapiro and Harper, 1999). Thus, the observation that overexpression of p21 protein was not sufficient to sustain a G1 arrest in amifostine-treated cells suggests that amifostine induced the expression of one or more other cell cycle inhibitors downstream of p53 protein.

In HCT116 cells, amifostine, a cytoprotective agent, also has the potential to induce apoptosis. In the presence of p53 protein, HCT116 cells exhibited low-level resistance to amifostine, indicating that p53 protein protected cells from amifostine-induced apoptosis. The degree of resistance to amifostine conferred by p53 protein was modest, in the range of 1.5–2-fold, and the biologic relevance of such relatively small degrees of resistance is poorly understood. Amifostine triggered apoptotic cell death at concentrations that are only slightly above the plasma concentrations achieved in clinical studies (Shaw *et al*, 1986,^1988^), indicating that this observation may have clinical relevance.

Taken together, our findings indicate that p53 protein plays an important role in regulating the cellular response to amifostine and identify p53 protein as a mechanism of resistance to amifostine-induced apoptosis and as a mechanism of amifostine-induced G1 arrest and cytoprotection.
